# Impact of the radiographic examination on diagnosis and treatment decision of caries lesions in primary teeth – the Caries Detection in Children (CARDEC-01) trial: study protocol for a randomized controlled trial

**DOI:** 10.1186/s13063-016-1196-5

**Published:** 2016-02-09

**Authors:** Fausto Medeiros Mendes, Laura Regina Antunes Pontes, Thais Gimenez, Juan Sebastian Lara, Lucila Basto de Camargo, Edgard Michel-Crosato, Claudio Mendes Pannuti, Daniela Prócida Raggio, Mariana Minatel Braga, Tatiane Fernandes Novaes, CARDEC collaborative group

**Affiliations:** Department of Pediatric Dentistry, School of Dentistry, University of São Paulo, São Paulo, Brazil; Department of Pediatric Dentistry, Universidade Paulista, São Paulo, Brazil; Departament of Community Dentistry, School of Dentistry, University of São Paulo, São Paulo, Brazil; Division of Periodontics, School of Dentistry, University of São Paulo, São Paulo, Brazil; Instituto de Odontologia, Universidade Cruzeiro do Sul, São Paulo, Brazil

**Keywords:** Dental caries, diagnosis, visual inspection, radiographic method, randomized clinical trial

## Abstract

**Background:**

Although most clinical guidelines throughout the world indicate that clinicians take two bitewings for detecting caries lesions in primary molars of all children, evidence for this recommendation is essentially based on cross-sectional studies performed in laboratory settings or using convenience samples. The benefits and impact of performing radiographs on diagnosis and treatment decision of caries lesions in primary teeth, mainly considering relevant outcomes for patients, have not been evaluated yet. Thus, the aim of this randomized clinical trial will be to evaluate the impact of performing radiographic examination adjunct to the visual inspection for detecting and making treatment decision regarding caries lesions in primary teeth compared with visual inspection performed alone. We will consider different outcomes related to children's health and welfare.

**Methods/Design:**

To reach this objective, 250 children ages 3 to 6 years who sought dental treatment in our dental school will be randomly allocated in two groups according to the diagnostic strategy used for caries detection: visual inspection performed alone or visual inspection associated to radiographic examination. Two trained and calibrated examiners will carry out the examinations and elaborate the treatment decision plan. Then, children will be treated and followed up for 2 years, with evaluations after 12 and 24 months after the inclusion of children in the study. Children will also return after 6 and 18 months to reinforce the preventive orientations. Primary outcome will be the number of dental surfaces in need of dental treatment at the follow-up. Secondary outcomes will be the components of the primary outcome separately, as well as, proportion of false-positive results, the oral health-related quality of life, cost-efficacy, cost-adjusted life years, and number of new lesions in the first permanent molars.

**Discussion:**

Our working hypothesis is that radiographic examination would actually exert little influence on patient-centered outcomes, and visual inspection would be enough as diagnostic strategy for caries detection in primary teeth.

**Trial registration:**

NCT02078453. Registered 4 March 2015.

**Electronic supplementary material:**

The online version of this article (doi:10.1186/s13063-016-1196-5) contains supplementary material, which is available to authorized users.

## Background

### Background

Visual inspection is a quick and easy method for caries lesions detection in primary teeth [1]. Moreover, the method has presented high specificity [2], and it is the unique method validated to assess caries lesions activity [3, 4]. For those reasons, it is routinely used in daily clinical practice [1].

Nevertheless, the method has not presented high sensitivity in detecting caries lesions, mainly at proximal surfaces [2]. To overcome this limitation of the visual inspection, many clinical guidelines used throughout the world have advised that dentists take two bilateral bitewing radiographs in children to detect missed caries lesions in primary molars [5–7]. The radiographic method is capable of increasing the sensitivity of visual inspection in primary teeth, decreasing the number of false-negative results in occlusal [2] and proximal surfaces [2, 8].

However, the increase in the sensitivity usually occurs with the expense of a higher number of false-positive results. Considering the dental caries, this increase of false positives may not be good for two main reasons:

1. The prevalence of non-evident caries lesions seems to be low in most populations. Thus, there would be a higher number or diagnostic errors using methods with low specificity than with low sensitivity;
2. A false-positive result would lead to an unnecessary operative treatment, whereas a false-negative result could be followed up and detected further with no consequences for the patient.

In addition, performing unnecessary operative treatment seems to be more costly than missing some caries lesions undetected by visual inspection alone [9].

Some studies have observed this trend of an increased number of false-positive results with the radiographic method [10–13]. However, most of these studies considered the criterion validity of the methods; hence, they compared the results of the methods with the results obtained with a reference standard method. This type of research usually obtained the rate or correct diagnosis of the method, but it is not concerned with the benefits for the patients.

No study performed to evaluate caries diagnosis strategies has ever evaluated important outcomes for the patients. A correct diagnosis is not necessarily a benefit for the patient. For instance, the detection of a caries lesion in a primary tooth near its exfoliation is not good for the patient because it would lead to unnecessary operative treatment. Therefore, studies that evaluate the benefits for the patients are essential to evaluate the actual utility of radiographic method to detect caries lesions in primary teeth. This reason motivates the realization of the present study.

### Objective

The aim of the present protocol will be to evaluate the effect of caries lesions detection in primary teeth performed with the radiographic examination adjunct to the visual inspection on the occurrence of outcomes related to the oral health of children through a randomized clinical trial.

### Trial design

A randomized controlled clinical trial with two parallel arms will be designed. One group will comprise children who will receive the diagnosis and treatment decision planned with the visual inspection alone. Another group will be formed by children who will receive diagnosis and subsequent dental treatment planning using visual inspection associated with radiographic examination.

## Methods

This article adheres to the guideline for randomized clinical trial protocols (SPIRIT). The SPIRIT checklist is described in the Additional file 1.

### Participants, interventions, and outcomes

#### Setting

The children will be randomly selected from a pool of enrollment forms of children (3 to 6 years old) who sought dental treatment at our school. Because we will select patients who looked for dental treatment, we can consider extrapolating the results to the dental office setting. This context is adequate since clinicians usually apply these diagnostic strategies in the daily clinical practice.

#### Eligibility: inclusion and exclusion criteria

The inclusion criteria will consider the following children:

1. who sought dental treatment in our school;
2. are aged 3 to 6 years old; and
3. have at least one primary molar in the mouth.

The following will be excluded from the study:

1. children whose parents refuse to participate in the research or
2. children who present behavioral problems during the initial appointments.

#### Interventions

An initial clinical examination will be performed to evaluate the teeth present in the mouth, as well as the caries experience using the World Health Organization criteria [14]. The children will be classified in subgroups according to age (3 and 4 years old or 5 and 6 years old) and according to the caries experience (children with decayed-missing-filled-surfaces (dmf-s) less than or equal to 3 or children with dmf-s greater than 3).

If the child is eligible to participate in the study, bitewing radiographs will be taken from each side, including the upper and lower primary molars (two bilateral radiographs for each child), as recommended by different clinical guidelines throughout the world [5–7]. Complementary periapical radiographs also will be taken when necessary. Radiographs will be processed using the time/temperature method, in which the film stays in the revealing solution (Eastman Kodak, Rochester, NY, USA) for 2 min at a temperature of approximately 27 °C [15], followed by a fixation time of 10 min in fixing solution (Eastman Kodak, Rochester, NY, USA), and then by washing in water for 20 min [15]. The revealing and fixing solutions will always be new, replaced at the beginning of each period of clinical appointments, guaranteeing the quality of processing.

In addition, a questionnaire to evaluate the impact of oral health on the quality of life of the children at baseline will be applied for the parents. The instrument used will be the Brazilian version of the Early Childhood Oral Health Impact Scale (ECOHIS) [16, 17]. Then, the participant will be randomly allocated in one of the two groups.

The groups will be defined according to the diagnostic strategy used for reaching the treatment decision related to dental caries in primary molars proposed for each child. The two groups are as follows:

1. Visual inspection alone: The treatment decision plan will be based only on a visual inspection. The examiners will not receive the bitewings of these children to elaborate the treatment plan. They will only access periapical radiographs when necessary (to decide between endodontics or tooth extraction, for example); those radiographs will be available after the decision regarding the process for the other teeth has been rendered.
2. Visual inspection plus radiographic: The treatment plan will be based on the visual inspection complemented by radiographic examination. The access to periapical radiographs is also permitted in this case.

At the second clinical appointment, two different examiners will perform the examination and elaboration of the treatment plan. They will be trained and calibrated prior to the study. During the study, their calibration will also be checked after each 50 children included in the study.

#### Caries detection procedures

The visual inspection will be done according to the International Caries Detection and Assessment System (ICDAS) [18]. The children will be positioned in a dental chair, under illumination, and they will receive prophylaxis using rotating bristle brush and a pumice/water slurry. The examiners will use a dental mirror and a ball-ended probe for the examination. The teeth will be examined wet, and then, they will be air-dried for 5 s with a 3-in-1 syringe. The examiner will also evaluate the caries activity status if a caries lesion is present [19]. The condition and treatment decision of each dental surface will be recorded on an appropriate form.

In all cases, the clinical evaluation will be performed without knowledge of the experimental group of the participant. After the examination, the examiner will be informed of the enrollment group and will plan the treatment with or without access to the bitewing radiographs.

For the children allocated to the experimental group, the same examination procedure will be done, but a visual inspection and radiographic examination will be used to reach the treatment decision. Radiographic evaluation will be performed using a light box.

The treatment plan elaborated according to the allocated group will be put in envelops that will be delivered for the dentists responsible for performing the dental treatment. On the first day of treatment, the children will receive orientation for oral hygiene and dietary advice, and an anamnesis will be performed with the parents of the children. Dentists will perform the dental treatment following the plan and according to the predetermined protocols for each type of treatment.

#### Dental treatment protocols

The choice of the protocols of treatment is based on the best available evidence:

1. Operatory treatments will be done with partial caries removal [20].
2. High-viscosity glass ionomer cement will be used to restore cavitated active caries lesions in occlusal [21] or approximal [22] surfaces (score 4 of ICDAS or higher and/or lesions reaching the outer half of the dentin in the radiographic image).
3. Resin-modified glass-ionomer cement will be used for restorations of lesions involving more than two surfaces [23].
4. Non-cavitated active caries lesions will be treated with fluoride varnish [24].
5. Prevention measure orientation will be based on the orientation of oral hygiene using fluoride dentifrice with 1000 to 1500 ppm of fluoride [25] and dietary advice [26].
6. Endodontic treatment will use iodoform paste [27, 28].
7. Dental extractions and other types of treatment will be provided.

The dentists responsible for the treatments will not receive the bitewings radiographs of the participants; they will only have access to periapical radiographs that may be useful for indirect pulp capping, endodontic treatment, and extraction.

During the operatory interventions, the presence of soft or hard carious tissue or the absence of carious tissue will be evaluated in order to record possible false-positive diagnosis for dentine caries.

The time required for all procedures and the materials used will be registered by an external examiner for the economic analysis. The time spent for each procedure, including return visits, will be considered in calculations of the direct and indirect costs.

The number of visits of each participant and the procedure done at each session, with their respective duration times, also will be recorded. For the calculation of direct costs, we will consider the average of market prices of the materials used in each procedure [29, 30]. Such values will be obtained by averaging the prices from different sources for the products being used, and these numbers will be updated during the study. For the calculations, indirect costs will be also considered, as described in previous studies [29, 30].

#### Follow-up visits

After the end of the dental treatments, the children will be recalled every 6 months to reinforce the preventive orientations concerning the diet and biofilm control. Furthermore, the participants will be orientated to contact us in case of a new complaint. In this case, the additional treatment will be immediately made and registered.

After 12 and 24 months, the number of dental surfaces with dental treatment will be determined for all participants. For this, two different examiners, blinded in relation to the experimental group of the children, will evaluate the conditions and the need for new operative interventions. They will record the following:

1. dental surfaces requiring operative treatment (evident dentine caries - cavitated or not);
2. restored dental surfaces requiring replacement (large failures, caries around restorations, and the complete loss of the material will be considered);
3. restored dental surfaces requiring repair (small failures); and
4. teeth requiring endodontic treatment or extraction (in both cases, summing five surfaces per tooth).

Children with treatment needs will be treated by one of our dentists. All children will receive hygiene and dietary instructions, and fluoride products will be applied as needed. After 24 months, the ECOHIS will be reapplied for the children.

#### Outcomes

The primary outcome will be the number of dental surfaces with operative treatment needs in the follow-up. This outcome is composed of several mutually exclusive factors: number of surfaces with new dentin caries lesions; number of restored surfaces with necessity of replacement; tooth with pain episode and/or necessity of endodontic treatment and tooth indicated for extraction.

The separate components of the primary outcome will be considered as secondary outcomes. Other secondary outcomes will be the impact of oral health on quality of life, number of false-positive results, number of new lesions in the first permanent molars, cost-efficacy and quality-adjusted life year.

#### Participant timeline

**Fig. 1:**
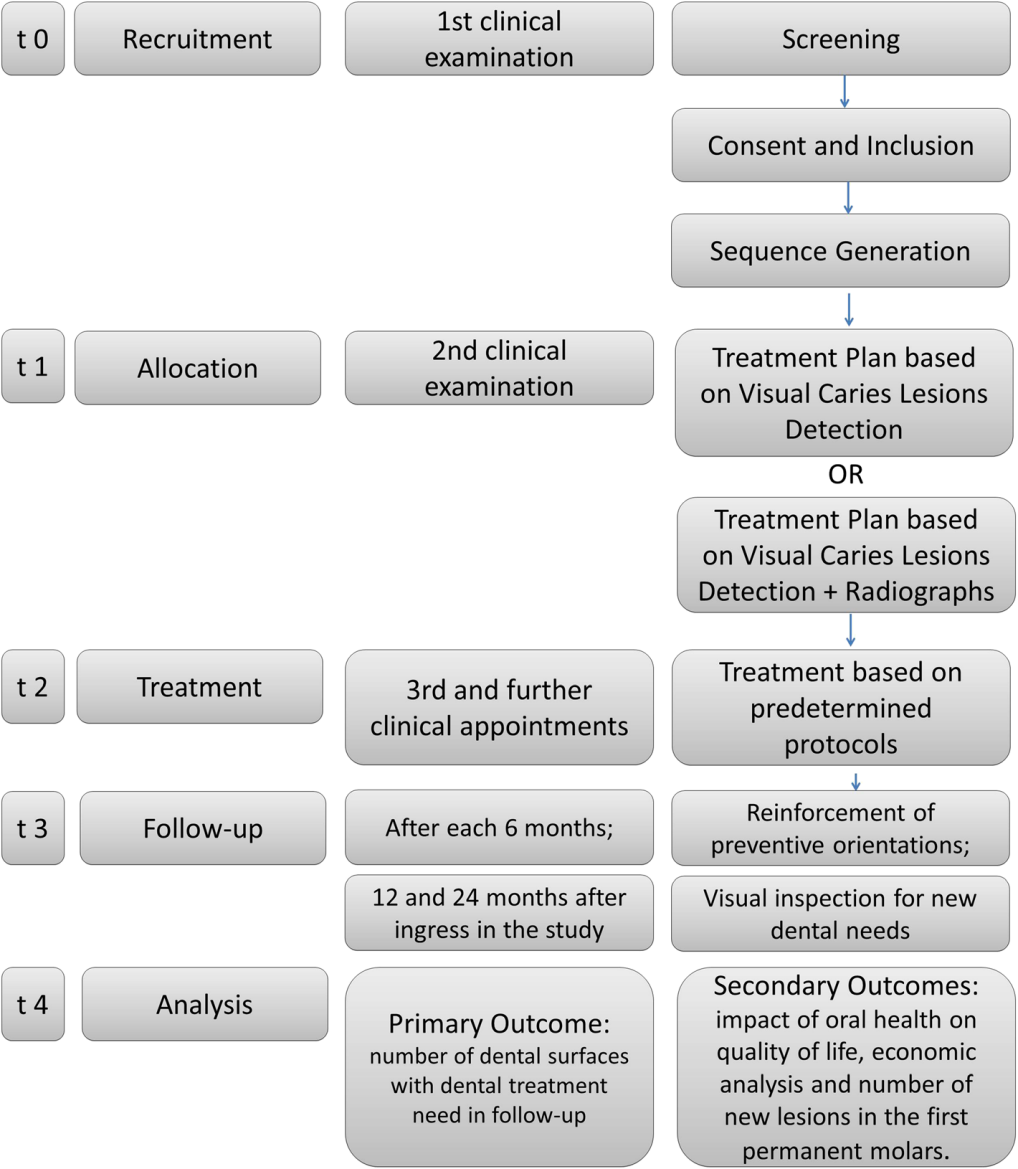
Timeline of the study procedures

Recruitment will take place from April 2014 to December 2015. Each participant will be enrolled in the study for approximately 25 months in total (1-month RCT – diagnosis and treatment, followed by a 24-month observational period). The timeline with details of the data collection schedule are summarized in Fig. 1.

#### Sample size

The sample size calculation was based on the occurrence of the primary outcome: number of dental surfaces of primary molars with operative needs during the follow-ups and the incidence data. Considering operative needs, we observed a mean of 17.6 surfaces with new caries lesions after 2 years [31], approximately 10 % restoration failure of occlusal or occluso-proximal restorations in 2 years [32], 0.08 extracted teeth in 2 years, with 0.2 surfaces [33] and 0.3 pain episodes in 2 years, with one surface [33].

Therefore, a mean of 19 surfaces with treatment need in the visual inspection alone group is expected. A difference of five surfaces with treatment need in the visual plus radiographic method group was considered as a minimally clinically important difference. The standard deviation values expected for visual and visual plus radiographic groups were 15 and 10, respectively. Therefore, using a two-tailed test and considering a significance level of 5 % and 80 % power, the minimum sample size of children calculated was 103 per group. Anticipating an attrition rate of 80 %, the final minimum sample size was 250 children for the entire study.

#### Recruitment

Recruitment is based in our School of Dentistry, which refers children who seek dental care.

### Assignment of interventions

#### Allocation: sequence generation

The participants will be selected from a pool of enrollment forms of children who looked for dental treatment in our school, using a sequence of random numbers generated by software. The randomization procedure will be done per blocks of the same size and stratified by age and caries experience groups.

#### Allocation concealment mechanism

We will use sealed, sequentially numbered, opaque envelopes, separated by each stratum. The randomization will be done after the inclusion of the child and after the radiographs. The group will be revealed for the examiners after the clinical examination.

#### Implementation

The examiner who performs the first clinical examination will see and designate the allocation of each child using the opaque envelops. Then, she will inform it only to the examiners who will perform the visual inspection and treatment plans.

#### Blinding (masking)

Children and their parents, as well as, the dentists responsible for the dental treatment, and the examiners who will evaluate the outcomes during the follow-up will be blinded regarding the allocation group.

### Data collection, management, and analysis

#### Data collection methods

Data collection and returning assessments will be made by researchers who have been trained to use ICDAS and to identify new dental treatment needs. They will be blinded to group allocation, and they will be the same examiners at all time-points for each participant in order to minimize inter-observer variability.

#### Data management

Clinical data will be entered directly onto predetermined sheets. Data quality will be ensured by validation checks that include missing data, out-of-range values, and illogical and invalid responses.

#### Statistical methods

For comparing the outcomes between the two groups, Student’s *t* test and Poisson regression analysis will be performed. With regard to the impact of Oral Health on quality of life, differences in the final and baseline scores will be compared between the groups through Student’s *t* test or the Mann–Whitney test, depending on the normality of the data distribution.

Multivariate analysis will be conducted to investigate the influence of the radiographic examination on treatment decision. Time and treatment cost will be compared by Student’s *t* test. An incremental cost-efficacy ratio will be used to compare the economic impact of both diagnostic strategies, considering both the initial examination and possible treatment and re-treatments during the study. The quality-adjusted life year (QUALY) will be also calculated in order to estimate the ratio of cost saved/spent by the use of the proposed diagnostic strategy. For all analyses, the level of significance will be set at 5 %.

### Monitoring

#### Data monitoring

As adverse events related to the detection of caries lesions and dental treatments are unlikely, no Data Monitoring Committee will be established, and independent oversight of trial data collection, management and analysis will be undertaken by one author (FMM). The chief investigator (FMM) has overall responsibility for the study and is custodian of the data.

#### Harms

It is unlikely that our procedures will result in any adverse effects beyond those listed as trial outcomes. These effects are usually expected in any conventional dental treatment performed in pediatric dentistry clinical practice.

#### Auditing

Data entered will be subjected weekly to audit by the coordinator, and data queries will be raised as necessary. Any discrepancies detected will be corrected and systematically registered.

### Ethics and dissemination

#### Research ethics approval

The present protocol was submitted and approved by the Ethical Committee of the School of Dentistry, University of São Paulo on 25 May 2012.

#### Consent or assent

The participants’ parents or guardians will receive and sign an informed consent prior to the child being included in the research. Only children whose parents sign the consent will participate in the study.

#### Confidentiality

Participant confidentiality will be ensured using identification code numbers. Participant identifiable information will be stored in locked filing cabinets in a secure room. Medical information may be given only to the dentist’s team.

#### Access to data

Data generated from this trial will be available for inspection by request to the coordinator.

#### Ancillary and post-trial care

After completing the study, participants will continue to receive dental treatments, if needed, in our dental clinics.

#### Dissemination policy

Results will be reported in full through peer-reviewed journals, patient newsletters and a website.

## Discussion

We expect this study to provide the best scientific evidence for defining better diagnostic strategies for use in detecting caries in primary teeth. Considering the research architecture in diagnosis [34], the diagnostic studies have basic designs with increasing level of evidence for answering four basic questions in diagnostic research. The first three basic questions are answered through cross-sectional studies for method validation. Studies that address Phase 3 questions are performed to test the method in the target populations selected, consecutively or randomly, reducing the chance of selection bias, which may overestimate the performance of the diagnostic methods [35]. Several cross-sectional studies of accuracy have been published evaluating different methods of caries detection [36–38]. Nevertheless, we observed that most studies are lacking in the evaluation of clinically relevant aspects or patient-centered outcomes [39].

We observed in a recent published study that the additional tests do not bring great benefits to detect carious lesions in primary molars [12]. Since the introduction of selection bias was minimized in this study, strong evidence exists with respect to the detection of caries in primary teeth.

However, randomized clinical trials evaluating relevant outcomes for patients (Phase 4 questions) represent a higher degree of evidence in diagnostic research. This type of study is conducted to evaluate if patients who undergo a diagnostic method fare better than untested patients [34]. As an example, we can cite the issue of mammography for breast cancer detection. The validity of mammography has been confirmed by cross-sectional studies that perform the biopsy as the gold standard [40]. However, it is known that the real benefit of performing mammography as a screening test in women between 40 and 50 years of age is small. This observation is because the test would prevent death from breast cancer in less than 0.01 % of women under age 50 who undergo screening. Considering the problems of unnecessary treatment due to false-positive results, stress caused by the diagnosis of women who do not die from this disease (correct and incorrect diagnoses) and other problems, the risks outweigh the benefits of mammography in this age group [41]. This type of results can be only evaluated in randomized clinical trials because the validity studies do not deal with this aspect.

Until now, however, no randomized clinical trial was conducted to evaluate caries diagnosis strategies. With the expected results, we aim to achieve the refutation of the recommendation to conduct bitewing radiographs for detecting caries lesions, even in children without signs or symptoms, which is present in all protocols of clinical procedures worldwide. On the other hand, in case of favorable results obtained with the experimental group, we will confirm the benefits of strategies of caries detection advised by those clinical guidelines. To the best of our knowledge, this is the first randomized clinical trial to evaluate diagnostic strategies for diseases related to the oral cavity, considering the whole playing field of dentistry.

## Trial status

**Fig. 2:**
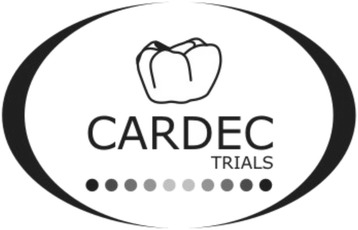
CARDEC trial logotype

This is an ongoing trial, which is still recruiting participants at this moment. Figure 2 presents the CARDEC trial logotype. The CARDEC collaborative group represents all persons involved at this trial or in other studies that are been conducted and are nested in the CARDEC-01 trial. The group is formed by researchers, dentists, graduate and undergraduate students and technicians. The detailed roles of each member and respective affiliations are described in Additional file 2.

At the time of the submission of this manuscript, 225 participants had been included. Final results are expected by the beginning of 2018.

## Additional files

Additional file 1:
**SPIRIT 2013 Checklist: Recommended items to address in a clinical trial protocol and related documents*.** (PDF 129 kb) Additional file 2:
***CARies DEtection in Children (CARDEC) collaborative group.** (PDF 11 kb)

## Abbreviations

CARDEC: Caries Detection in Children
ICDAS: International Caries Detection and Assessment System
